# Naturally Occurring Variants of Human Α9 Nicotinic Receptor Differentially Affect Bronchial Cell Proliferation and Transformation

**DOI:** 10.1371/journal.pone.0027978

**Published:** 2011-11-18

**Authors:** Anna Chikova, Sergei A. Grando

**Affiliations:** 1 Department of Dermatology, University of California Irvine, Irvine, California, United States of America; 2 The D.I. Ivanovsky Institute of Virology of The Ministry of Health of The Russian Federation, Moscow, Russia; 3 Cancer Center and Research Institute, University of California Irvine, Irvine, California, United States of America; Instituto Nacional de Câncer, Brazil

## Abstract

Isolation of polyadenilated mRNA from human immortalized bronchial epithelial cell line BEP2D revealed the presence of multiple isoforms of RNA coded by the CHRNA9 gene for α9 nicotinic acetylcholine receptor (**nAChR**). BEP2D cells were homozygous for the rs10009228 polymorphism encoding for N442S amino acid substitution, and also contained mRNA coding for several truncated isoforms of α9 protein. To elucidate the biologic significance of the naturally occurring variants of α9 nAChR, we compared the biologic effects of overexpression of full-length α9 N442 and S442 proteins, and the truncated α9 variant occurring due to a loss of the exon 4 sequence that causes frame shift and early termination of the translation. These as well as control vector were overexpressed in the BEP2D cells that were used in the assays of proliferation rate, spontaneous *vs.* tobacco nitrosamine 4-(methylnitrosamino)-1-(3-pyridyl)-1-butanone (**NNK**)-induced cellular transformation, and tumorigenicity in cell culture and mice. Overexpression of the S442 variant significantly increased cellular proliferation, and spontaneous and NNK-induced transformation. The N442 variant significantly decreased cellular transformation, without affecting proliferation rate. Overexpression of the truncated α9 significantly decreased proliferation and suppressed cellular transformation. These results suggested that α9 nAChR plays important roles in regulation of bronchial cell growth by endogenous acetylcholine and exogenous nicotine, and susceptibility to NNK-induced carcinogenic transformation. The biologic activities of α9 nAChR may be regulated at the splicing level, and genetic polymorphisms in CHRNA9 affecting protein levels, amino acid sequence and RNA splicing may influence the risk for lung cancer.

## Introduction

The functional components of cholinergic system are highly conserved among the invertebrate and vertebrate species. Previous genome wide association studies revealed single nucleotide polymorphisms (**SNPs**) in the CHRNA5-CHRNA3-CHRNB4 gene cluster on chromosome 15 that encodes the α5, α3 and β4 subunits of the nicotinic acetylcholine receptors (**nAChRs**) associated with nicotine dependence and lung cancer [1], [2], [3]. However, the majority of these SNPs do not affect protein sequences, because they are located in non-coding regions or result in synonimous codon substitutions. The genetic variations in other nAChR subunits expressed in lung epithelia [4], [5], [6], [7] remain largely unknown.

In the past, we have established an important role for nAChRs expressed on respiratory cells in the malignant transformation caused by pharmacological doses of tobacco-derived carcinogenic nitrosamines, and demonstrated that nicotinic antagonists can abolish these pathobiologic effects [8]. Nicotine and its carcinogenic derivatives can contribute to the development of lung cancer by acting as tumor promoters that facilitate the outgrowth of cells with genetic damage [9]. In contrast, inhibition of nAChRs induces apoptosis of lung cancer cells and produces anti-carcinogenic effects [10], [11], [12]. Therefore, pulmonary nAChRs are now widely considered as novel drug targets for prevention and treatment of lung cancer [13], [14], [15].

We focused this study on the CHRNA9 gene encoding α9 nAChR originally cloned by us from human epidermal keratinocytes [16]. Recent publications have demonstrated overexpression and activation of the α9 nAChR in human breast epithelial cells during tumorigenesis [17], and antitumor effects of inhibition of α9 signaling in human breast cancer cells [18], [19].

In this study, we evaluated effects of the naturally occurring variants of α9 nAChR in the cellular proliferation and neoplastic transformation. The results demonstrated dramatic variations in the biologic effects of α9 variants on human bronchial cells. These findings suggest a role of α9 nAChR in lung cancer development and progression.

## Methods

### Ethic Statement

All animal work have been conducted according to relevant national and international guidelines. The protocol of animal experiments number 2007–2732 was approved by the University of California Irvine Institutional Animal Care and Use Committee.

### Expression of CHRNA9 variants

The BEP2D cell line was a kind gift from Dr. Harris (NCI, NIH). This human papillomavirus-immortalized human bronchial epithelial cell line is non-tumorigenic, anchorage-dependent and sensitive to contact growth inhibition [20]. The cells were grown in EpiLife® medium containing 60 µM calcium and Human Corneal Growth Supplement (Invitrogen, Carlsbad, CA) in a humidified incubator at 5% CO_2_ and 37°C. Total RNA was purified from BEP2D cells using RNA purification kit (Qiagen, Valencia, CA). The SuperScript III First-Strand Synthesis SuperMix (Invitrogen) was used for cDNA synthesis with oligo(dT) primer. cDNA was amplified using the Platinum PCR SuperMix High Fidelity (Invitrogen), and the primers 5′ GATGAACTGGTCCCATTCCTG and 5′ GATAGCAAAGCCAATATCTGTG. The oligonucleotides 5′GAATGCTGCGGCCGCATGAACTGGTCCCATTCCTGCATC and 5′CTCTGCTACGCGTAGCCAATATCTGTGACTAATCC were used for the second round of PCR and preparation of the cDNA fragment for cloning. The CHRNA9 open reading frame was inserted into pLVX-tight-Puro plasmid (Clontech, Mountain View, CA) by NotI/MluI restriction sites. The N442 variant of the protein was prepared by site-directed mutagenesis with the use of QuikChange II XL Site-Directed Mutagenesis Kit (Agilent Technologies, Inc. Santa Clara, CA) and oligonucleotides 5′GACCACAAGGCCACCAATTCCAAGGGGAGTG and 5′CACTCCCCTTGGAATTGGTGGCCTTGTGGTC. Lentiviral particles containing CHRNA9 expressing pLVX vectors and non-expressing control vector were mixed with pLVX-Tet-off lentivirus and used for transduction of BEP2D cells following the manufacturer's protocol. Recombinant cells were selected and maintained in presence of 50 µg/ml of Geneticine (G418) and 5 ng/ml of Puromycin (Invitrogen). Antibiotic-resistant cells were maintained for less than 4 passages prior to experiments.

### Quantitative real-time PCR (qPCR)

RNA was purified from the exponentially growing cells and treated by RNase-Free DNase (Qiagen), followed by preparation of cDNA, as described above. Platinum SYBR Green qPCR Super Mix-UDG with ROX (Invitrogen) was used for qPCR with 100 µg of cDNA and the following primers: the full-length mRNA specific primers 5′CTTATATAACAAGGCTGATGATG and 5′ CAAAAGTCAGGTTGCACTG; the 122 amino acid (**aa**) variant specific primers 5′ CATCGTCTTATATAACAAGTAAATAC and 5′GGTTAGACTCTGGGAGTTTG; and the primers common for both variants of CHRNA9 cDNA 5′CTCTCTCAGATTAAGGATATGG and 5′ CTAGGCCATCGTACTGATC. The following primers specific for human ubiquitin C gene were used as loading control: 5’GAACGCCGATGATTATATAAG and 5′CATTGTCAAGTGACGATCAC. Quality of each reaction was controlled by dissociation curves and 2% agarose gel electrophoresis. Results of all qPCR reactions, performed with use of 7500 Real-Time PCR System (Applied Biosystems, Carlsbad, Ca), were adjusted by corresponding loading control data. Standard curve including multiple dilutions of the positive control plasmid was used to convert Ct-values into fold-difference for each pair of primers. One-way ANOVA with the Bonferroni post-test was used for statistical analysis.

### Cell growth and transformation assays

Exponentially growing BEP2D cells were plated on multiple 60 mm dishes at a density of 5×10^4^ cells per dish. The cells were counted in replica plates every 48 h using Cellometer Auto T4 (Nexcelom, Lawrence, MA), and the results of 4 independent experiments were analyzed. To determine susceptibility to carcinogenic transformation, BEP2D cells were exposed to 2 µg/ml of 4-(methylnitrosamino)-1-(3-pyridyl)-1-butanone (**NNK**; Toronto Research Chemicals Inc., Canada) or to mock for 48 h, put through 5 subsequent passages, and used in the soft agar anchorage-independent growth assay [8], [21], [22] and focus-formation assay [21], [22]. All experiments were performed in antibiotic-free media. In the anchorage-independent growth assay, multiple dilutions of test BEP2D cell lines in the media containing 0.3% of agar placed on the solidified layer of 0.5% agar were incubated for 14 days, stained with 0.01% crystal violet in 70% methanol, and the colonies containing at least 50 cells were counted with the aid of dissection microscope. In the focus-formation assay, the cells were grown in T-25 flasks for 14 days with changing medium every 3 days, washed and stained by 0.05% crystal violet in 80% methanol. The foci were counted in three 1 cm^2^ randomly selected fields in each flask.

### Tumorigenesis assay

The cells were treated by NNK and seeded into soft agar, as described above. Colonies grown for 2 weeks in soft agar were randomly selected, picked with the aid of the dissection microscope and plated in a regular growth medium. Cell colonies were cultivated for two passages and the transformed clones were propagated in the RPMI medium (Invitrogen) containing 10% of heat-inactivated FBS and Antibiotic-Antimycotic (Invitrogen). Anchorage-independent colonies are resistant to FBS-induced terminal transformation [23]. Approximately 2×10^7^ cells from each colony were injected subcutaneously to 4 weeks old male SCID mice (NOD.Cg-*Prkdc^scid^ Il2rg^tm1Wjl^*/SzJ, The Jackson Laboratory, Sacramento, CA). Each mouse received 4 different cell clones, one per each of the bilateral front and rear trunkal locations. Mice were monitored weekly for the appearance of tumors and euthanized 2 months post-injection, and visible tumors were harvested and examined microscopically. These experiments were performed blindly with regard to the type of injected BEP2D cell clones.

### Statistical analysis

All statistical analyses were performed using the “Prism” software (GraphPad Software Ink., La Jolla, CA). One-way ANOVA with the Bonferroni post-test was used for analysis of allele-specific qPCR data. Two-tailed paired t-test was used for analysis of cellular proliferation, and two-tailed non-parametric test was used for analysis of cellular transformation. Two-tailed Fisher's exact test was used in the tumorigenesis assay. In all experiments, differences were deemed significant if the *p* value calculated with 95% confidence interval was less than 0.05.

## Results

### Naturally occurring variants of α9 nAChR expressed in BEP2D cells

**Figure 1:**
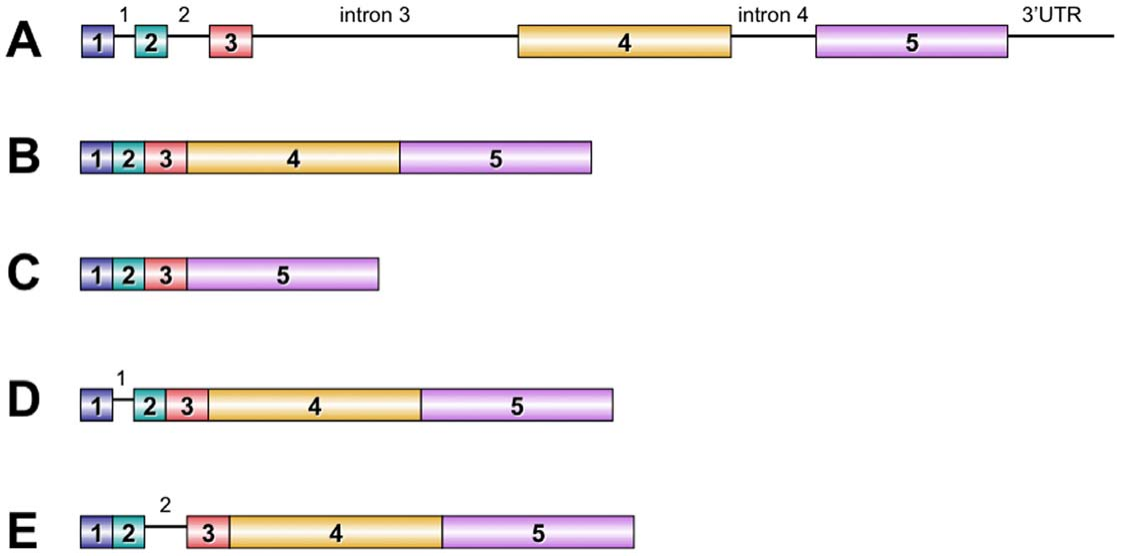
Structure and splicing of human CHRNA9 gene. **A**. Structure of the human CHRNA9 gene. Exons are shown as rectangles and introns are shown as lines. **B, C, D, E**. Variants of the splicing of mRNA coded by the CHRNA9 gene detected in BEP2D cells. The mRNAs for full-length (**B**) and truncated proteins (**C, D, E**) expressed in BEP2D cells. The truncated α9 used in overexpression experiments resulted from a loss of the exon 4 that causes frame shift with 122 aa peptide translation (**C**).

**Figure 2:**
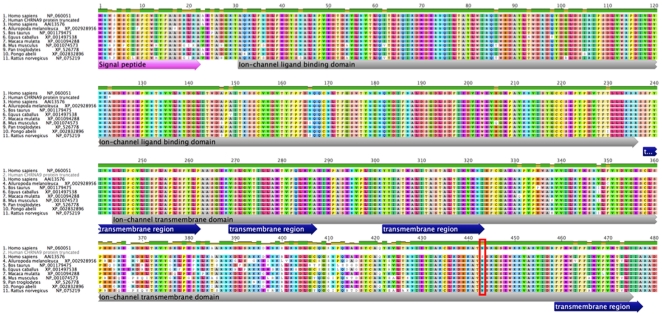
Alignment of α9 nAChRs from various mammals. Predicted functional domains are depicted by arrows. Alignment and annotations were generated using the Geneious software (Biomatters Ltd, Auckland, New Zealand). Protein sequences were obtained from the NCBI database. Asterisk indicates end of the 122 aa peptide translation. The N442S aa substitution region is highlighted by the red frame. Due to the gap in the alignment this aa appeared at position 443.

Polyadenilated CHRNA9 mRNA was cloned from BEP2D cells into pLVX vector. Sequence of resulting plasmids revealed presence of four isoforms of α9 mRNA (**Fig. 1**). The BEP2D cell line was found to be homozygous for the rs10009228 polymorphism and express an α9 nAChR protein with the N442S aa substitution. In addition to the normally spliced CHRNA9 mRNA, one of the isoforms had remaining intron 1 and another one had intron 2 sequences. These two isoforms may lead to frame-shifts and manifestation of the termination codon after aa 41 and 75, respectively. They were previously found in trachea and embryonic stem cells and are registered in the NCBI database (http://www.ncbi.nlm.nih.gov/IEB/Research/Acembly/av.cgi?exdb=AceView&db=36a&term=CHRNA9). The newly discovered fourth mRNA isoform resulted from the compete loss of the exon 4. This RNA splicing translates into the truncated 122 aa peptide that includes the 25 aa signal peptide and a part of the predicted neurotransmitter gated ion-channel ligand binding domain, but not the predicted transmembrane domain (**Fig. 2**).

### Overexpression of α9 nAChR variants in BEP2D cells

**Figure 3:**
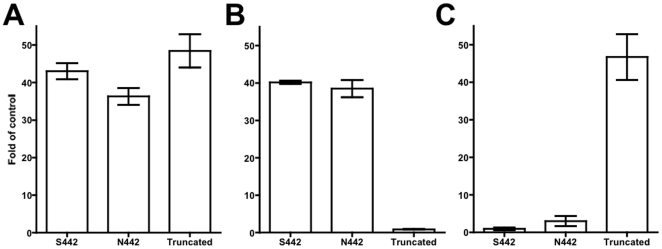
Overexpression of α9 nAChR variants in BEP2D cells. The relative amounts of mRNA encoding the variants of α9 nAChR overexpressed in BEP2D cell lines were measured by qPCR, as detailed in Methods, using the primers common for all α9 isoforms (**A**), specific for the full length α9 (**B**), and specific for the 122 aa truncated α9 variant (**C**). Data are means ± SEM of qPCR experiments performed with at least 3 biological replicates of each cell line and expressed as fold of pLVX control, taken as 1. One-way ANOVA with the Bonferroni post-test was used for analysis of allele-specific qPCR data.

The biologic significance of α9 variants was evaluated in BEP2D cells overexpressing the reference sequence of α9 containing N442 aa (NP_060051; prepared by the site-directed mutagenesis), the α9 containing S442 aa substitution (rs10009228; expressed in intact BEP2D cells) and the truncated 122 aa α9 peptide *vs.* the non-expressing vector pLVX (negative control). The resultant BEP2D cell lines were named by the α9 nAChR variant they overexpressed, i.e., "N442," "S442," “truncated” and "pLVX". Allele-specific qPCR assays confirmed significant (p<0.05) overexpression in experimental BEP2D cells of all three experimental α9 target mRNA, in comparison to the negative control cells (**Fig. 3**).

### Variants of α9 nAChR differentially influence proliferation and neoplastic transformation of BEP2D cells

**Figure 4:**
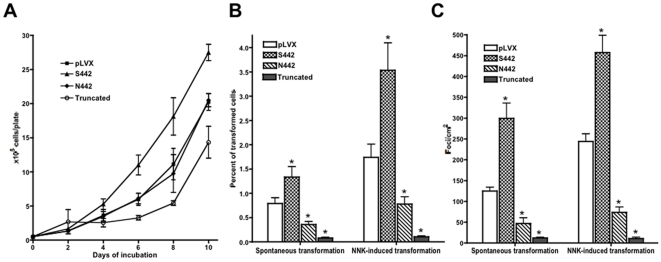
Differential effects of α9 nAChR variants on cell growth and neoplastic transformation. **A**. Growth curves. Total number of cells per a 6-well plate for BEP2D cells overexpressing N442, S442 or truncated α9 and control cells infected with empty pLVX vector. The data are means ± SEM obtained in 4 independent experiments. **B, C**. Cellular transformation assays. The BEP2D cells overexpressing N442, S442 or truncated α9 and control cells infected with empty pLVX vector were incubated for 48 h without (spontaneous transformation) or with 2 µg/ml NNK (NNK-induced transformation) and analyzed in soft agar (**B**) or focus formation (**C**) assay, as detailed in Methods. The data are means ± SEM obtained in at least 3 independent experiments. Asterisks  = p<0.05 compared to pLVX.

The S442 overexpressing BEP2D cells demonstrated significant (p<0.05) increase in the proliferation rate, compared to other cell lines (**Fig. 4A**). No difference between the growth rates of N442 and control cells was seen (p>0.05). In contrast, the BEP2D cells expressing truncated variant of α9 had a significantly (p<0.05) decreased growth rate.

The effect of α9 variants on spontaneous and NNK-induced cellular transformation was tested in both anchorage-independent growth (**Fig. 4B**) and focus formation (**Fig. 4C**) assays. Soft agar assay measures the fraction of cells that gained ability to grow without attachment to surface. Focus formation assay allows to visualize cells that lost contact growth inhibition and started to grow in multiple layers. Both assays brought similar results demonstrating dramatic differences among tested BEP2D cell lines. Overexpression of S442 α9 led to a significant (p<0.05) increase in both spontaneous and NNK-induced cellular transformation. In marked contrast, the N442 α9 variant significantly (p<0.05) reduced both spontaneous and NNK-induced transformation, compared to the control cells containing an empty vector. Overexpression of truncated α9 variant also led to a significant (p<0.05) decrease in both spontaneous and NNK-induced cellular transformation, compared to control. Notably, NNK-treatment caused no significant increase in the transformation of BEP2D cells overexpressing the 122 aa variant. These results demonstrated for the first time that overexpression of the truncated α9 variant protects BEP2D cells from the carcinogenic transformation caused by NNK.

### *In vivo* tumorigenesis

To confirm that results of our *in vitro* assays indeed represented tumorigenesis, we isolated single transformed BEP2D cell clones from the soft agar for expansion and subsequent injection to SCID mice. We isolated a total of 24 transformed colonies from each cell line growing in soft agar, and cultivated them for two passages in the EpiLife® medium used for maintaining BEP2D cell lines. Then, the cells were fed with RPMI medium containing 10% of heat inactivated FBS and expanded through three additional passages. It has been previously shown that anchorage-independent colonies obtained from BEP2D cells are resistant to FBS-induced terminal differentiation and survive this treatment [23]. Recovery of the transformed clones originated from N442, S442 and pLVX cell lines was at least 20 clones for each cell line, or 83%. In marked contrast, only 6 of 24 clones, or 25%, isolated from the BEPD2D cell line overexpressing truncated α9 protein were resistant to treatment with FBS. Such a low yield of viable clones indicated that overexpression of truncated α9 dramatically increases resistance to NNK-induced cellular transformation. To avoid spurious results due to huge differences in cell survival compared to other cell lines, the clones representing overexpression of truncated α9 variant were not used in *in vivo* experiments.

Transformed clones originated from N442, S442 and pLVX cell lines were injected subcutaneously to SCID mice. The morphological and histological analyses performed 2 months after inoculation revealed differences among their tumorigenic potential. Fisher's exact test demonstrated a significant (p<0.05) increase in tumor formation by the S442 overexpressing BEP2D cells (**Table 1**). This finding indicated that S442 variant of α9 nAChR not only elevates cellular transformation, but also promotes tumorigenicity of transformed clones.

**Table 1:** Tumorigenicity in SCID mice of NNK-transformed clones of BEP2D cell lines isolated from soft agar.*

Cell line	Total number of clones injected	Number of tumors	% of tumorigenic clones	P value (compared to pLVX)
pLVX	18	4	28	
S442	22	15	69	0.012
N442	20	2	10	0.4

*Each clone was introduced *via* a separate injection, as detailed in Methods. Tumorigenicity of S442 and N442 clones was compared to that of the BEP2D cells containing empty pLVX vector in two-tailed Fishers exact test with 95% confidence interval.

## Discussion

In this study, we demonstrated for the first time that different variants of human α9 nAChR subunit protein differentially influence regulation of bronchial cell proliferation and susceptibility to the tumorigenic transformation by NNK. Several normal human polymorphisms affecting aa sequence of α9 nAChR are reported in the NCBI database. Surprisingly, while the reference sequence of α9 nAChR NP_060051 contains N442 aa, available population data indicate that the reference allele has average frequency only around 0.2, while frequency of the rs10009228 SNP encoding the S442 α9 variant is approximately 0.8 (http://www.ncbi.nlm.nih.gov/projects/SNP/snp_ref.cgi?rs=10009228). Most importantly, our recent case-control study has demonstrated a significant association of the N442 α9 nAChR variant with a decreased risk of lung cancer [24].

Overexpression in BEP2D cells of the most frequent α9 population variant S442 promoted proliferation and facilitated NNK-induced tumorigenesis. An overexpression and activation of α9 in breast epithelial cells has been observed during tumorigenesis [17]. Further, inactivation of α9 signaling can inhibit proliferative, angiogenic and metastatic effects of nicotine/smoking [15], [18], [19]. The fact that S442 stimulated both cellular proliferation and tumorigenic transformation, suggested that the most common α9 nAChR variant acts as a tumor promoter. The tumor promoting activity may result from an increase in the proliferation rate and/or facilitation of genomic instability.

Suppression of cellular transformation by the N442 α9 variant and relatively rare occurrence of this ancestral protein in human population prompted further analysis. Based on the protein sequence alignment of the CHRNA9 homologs expressed in various mammalian species (**Fig. 2**), some parts of C-terminal domain display a large level of variability, but the aa spanning the 428–462 region are conserved among various species. Interestingly, while the majority of mammals carry the N442 variant, humans most commonly feature the S442 variant. Noteworthy, previous characterization of the ligand binding profile of α9 nAChR in *Xenopus oocytes*, which revealed an unusual response to the canonical agonists such as nicotine [25], was performed using the rat α9 nAChR that contains N442 residue. Our results demonstrating opposite effects of N442 and S442 variants on cell growth and resistance to oncogenic transformation suggest that the two variants may vary in their response to ligands, which needs to be elucidated in a separate study.

The results of the present study also demonstrated for the first time that overexpression of the truncated α9 variant increases resistance of BEP2D cells to carcinogenic transformation caused by NNK. Previously, we have reported that inhibition of acetylcholine signaling in BEP2D cells significantly reduces NNK-associated cellular transformation [8]. Therefore, it can be postulated that function of CHRNA9 gene in the acetylcholine signaling axis can be physiologically regulated at the level of RNA splicing.

Various isoforms of CHRNA9 mRNA are detected in different cell and tissue types (http://www.ncbi.nlm.nih.gov/IEB/Research/Acembly/av.cgi?exdb=AceView&db=36a&term=CHRNA9). Two CHRNA9 isoforms are expressed in cochlear hair cells of the chick [26]. The epigenetic regulation of cholinergic function by means of mRNA splicing has been characterized in the lower species that possess a smaller number nAChR genes, compared to humans. The posttranscriptional RNA modifications diversify the nAChR in insects [27], [28], [29]. In lower species, the RNA splicing may result in truncated nAChR proteins that can alter ligand binding charetristics [30] and mediate resistance to toxins [31]. The alternative splicing has been also reported for various mammalian nAChR genes, and can be associated with human diseases. For example, alternative splicing in the neuronal α4 and α7 nAChR subunits is associated with Tourette syndrome [32] and schizophrenia [33], respectively, and that of the α1 and ε subunits of the muscle-type nAChR—with congenital myasthenic syndromes [34], [35], [36]. Indeed, the presence of non-functional α1 nAChR resulting from RNA splicing was found in muscle cells [37]. Therefore, we hypothesize that expression of different isoforms of α9 nAChR protein affects cell cycle regulation in response to the endogenous ligand acetylcholine.

In conclusion, we demonstrated that different variants of the human α9 nAChR subunit protein differentially affect proliferation and neoplastic transformation of bronchial cells. Elucidation of the mechanisms, by which individual genetic variations in CHRNA9 affecting its expression, protein structure, and RNA splicing influence predisposition to lung cancer may lead to the development of personalized approaches to cancer prevention and treatment.
